# Arterial Spin Labeling Perfusion Study in the Patients with Subacute Mild Traumatic Brain Injury

**DOI:** 10.1371/journal.pone.0149109

**Published:** 2016-02-12

**Authors:** Che-Ming Lin, Ying-Chi Tseng, Hui-Ling Hsu, Chi-Jen Chen, David Yen-Ting Chen, Feng-Xian Yan, Wen-Ta Chiu

**Affiliations:** 1 Department of Diagnostic Radiology and Brain and Consciousness Research Center, Taipei Medical University Shuang-Ho Hospital, New Taipei City, Taiwan; 2 Graduate Institute of Injury Prevention and Control, Taipei Medical University, Taipei, Taiwan; 3 Department of Radiology, School of Medicine, College of Medicine, Taipei Medical University, New Taipei City, Taiwan; 4 Chia-Yi Hospital, Ministry of Health and Welfare, Chiayi City, Taiwan; University Medical Center (UMC) Utrecht, NETHERLANDS

## Abstract

**Background:**

This study uses a MRI technique, three-dimension pulse continuous arterial spin labeling (3D-PCASL), to measure the patient’s cerebral blood flow (CBF) at the subacute stage of mild traumatic brain injury (MTBI) in order to analyze the relationship between cerebral blood flow and neurocognitive deficits.

**Objective:**

To provide the relationship between cortical CBF and neuropsychological dysfunction for the subacute MTBI patients.

**Methods:**

After MTBI, perfusion MR imaging technique (3D-PCASL) measures the CBF of MTBI patients (n = 23) within 1 month and that of normal controls (n = 22) to determine the quantity and location of perfusion defect. The correlation between CBF abnormalities and cognitive deficits was elucidated by combining the results of the neuropsychological tests of the patients.

**Result:**

We observed a substantial reduction in CBF in the bilateral frontal and left occipital cortex as compared with the normal persons. In addition, there were correlation between post concussive symptoms (including dizziness and simulator sickness) and CBF in the hypoperfused areas. The more severe symptom was correlated with higher CBF in bilateral frontal and left occipital lobes.

**Conclusion:**

First, this study determined that despite no significant abnormality detected on conventional CT and MRI studies, hypoperfusion was observed in MTBI group using 3D-PCASL technique in subacute stage, which suggested that this approach may increase sensitivity to MTBI. Second, the correlation between CBF and the severity of post concussive symptoms suggested that changes in cerebral hemodynamics may play a role in pathophysiology underlies the symptoms.

## Introduction

Traumatic brain injury (TBI) is an important cause of disability and death in young adults. The majority (80–90%) of traumatic brain injury is classified as mild head injury [1–5] and the most mild traumatic brain injury (MTBI) patients recover within weeks to months but some patients may experience disabling symptoms, such as headaches, dizziness, memory problems and difficulties with learning new tasks [6], that interfere with their daily life. There is an increasing awareness of an epidemiologically large but silent presence of MTBI as well as its serious, long-lasting consequences in the recent years. In addition, biological markers of MTBI are still missing and some imaging evidence exists for the organic origin of the cognitive impairment.

Transient loss of consciousness or a brief amnestic, period with disabling physical (headache, dizziness, fatigue, noise and light sensitivity), cognitive (memory, attention, concentration, executive function deficits), and emotional/behavioral (depression, anxiety, irritability) symptoms lasting for months or years [7] is frequent among MTBI patients despite the absence of detectable damage on conventional MR imaging but correlated with the perfusion abnormality on imaging studies, mostly with single photon emission CT (SPECT) [8–13]. These studies demonstrated perfusion abnormalities, mostly hypoperfusion, predominantly in the frontal and temporal lobes, basal ganglia, and thalami, and to a lesser extent in the occipital lobes [9, 10]. Little literature focusing on the cerebral perfusion at the subacute stage of MTBI showed thalamic hypoperfusion [13]. Although most SPECT studies have revealed hypoperfusion in MTBI patients, the relationship between the cerebral blood flow (CBF) of cerebral cortex and neuropsychological impairment remained no man’s land.

Numerous imaging technique have been developed to evaluate cerebral hemodynamics, including positron emission tomography (PET), single photon emission computed tomography (SPECT), Xenon-enhanced computed tomography (XeCT), MRI dynamic susceptibility contrast (DSC), arterial spin labeling (ASL) and Doppler ultrasound. Among these techniques, ASL perfusion MRI, using blood water as an endogenous freely diffusible tracer, can provide absolute CBF value in the region of interest, which is great advantage over contrast agent based methods and is totally free of radiation. Besides, ASL MRI yields high resolution images without distortion artifacts [14].

The purpose of this study was to identify hypoperfusion areas using ASL MRI in MTBI patients by comparison with normal controls. In addition, we attempted to define the relationship between cortical CBF and neuropsychological disability in subacute MTBI patients.

## Materials and Methods

### Ethic statement

This study was approved by the Medical Research Ethics Committee of the Taipei Medical University, granted TMU-JIRB No. 201302027. All participants provided written informed consent. Between October 2010 and March 2012, 23 MTBI patients were included.

### Patient collection

Consecutive patients with MTBI from our three affiliated hospitals were prospectively identified for enrollment in this study through emergency department. The following criteria were used. (a) an age of 17 older; (b) MTBI, as defined by the American Congress of Rehabilitative Medicine; and (c) findings of a healthy brain at CT. The diagnosis of MTBI was established by using criteria of the American Congress of Rehabilitative Medicine for mild traumatic brain injury as follows: loss of consciousness less than 30 minutes, Glasgow Coma Scale (GCS) score of 13–15, and posttraumatic amnesia less than 24 hours. Patients were excluded if they had a history of epilepsy, cerebrovascular disease, intellectual disability, neurodegenerative disorders, prior TBI, important systemic medical illness, left-handedness, current use of psychoactive medications, or dental appliances that might distort the functional MR images. All the patients received the MRI and neuropsychological study within one month (12.57 ± 4.13 days) after MTBI. As a control group, age- and gender-matched healthy volunteers were recruited from a staff of hospital coworkers or volunteers through advertisement and were screened for neurologic, medical, and any psychiatric illness and left-handedness. All participants were administered a digit span (DS) and continuous performance test (CPT) outside the imager. DS is a short-term memory test measuring how many numbers a participant can remember in sequence. The CPT measures a person’s sustained and selective attention and impulsivity. The postconcussion syndrome (PCS) score was recorded in all patients. The post-concussion syndrome is defined as the presence of the following symptoms: headache, tiredness, dizziness, tinnitus, loss of concentration, depression, sleep disturbance, memory impairment, disorientation, irritability, and anxiety. The presence of each symptom scores one point in each patient and the total points a patient have is defined as the post-concussions syndrome (PCS) score. Other neuropsychological tests included the dizziness handicap index (DHI), simulator sickness questionnaire (SSQ), Beck anxiety inventory (BAI) and Wisconsin card sorting test (WCST). In the clinical assessment of the Wisconsin card sorting test, several functions of the frontal lobe such as strategic planning, organized searching, utilizing environmental feedback to shift cognitive sets, directing behavior toward achieving a goal, and modulating impulsive responding could be assessed from 6.5 years to 89 years of age. In our study, we focused on the perseverative errors in WCST only.

### MRI acquisition

Imaging data were acquired using a 3 Tesla GE whole-body scanner (Discovery MR750; GE Healthcare Systems, Milwaukee, Wisconsin) equipped with an 8-channel receive-only head coil. A high-resolution T1-weighted anatomical image was acquired using a three-dimensional fast spoiled gradient echo sequence (repetition time (TR) / echo time (TE) / inversion time (TI) = 8.2 ms / 3.2 ms / 450 ms, flip angle = 12°, field of view (FOV) = 240 x 240 mm^2^, matrix size = 256 x 256 x 176, slice thickness = 1 mm, covering the whole brain). The three-dimension pulse continuous arterial spin labeling (3D-PCASL) perfusion imaging was performed using a 3D background suppressed fast-spin-echo stack-of-spiral readout module with eight in-plane spiral interleaves (TR / TE / TI = 5327 ms / 10.5 ms / 2525 ms, labeling duration = 1500 ms, post-labeling delay = 2525 ms, no flow-crushing gradients, FOV = 240 x 240 mm^2^, in-plane matrix = 128 × 128, number of excitation = 4, slice thickness = 4 mm) and an echo train length of 36 to obtain 36 consecutive axial slices.

### Data Analysis

Quantification of cerebral blood flow (CBF) was obtained using the following equation, which was based on Functool software (version 9.4, GE Healthcare Systems).

$$\text{CBF}=6000\;\cdot \;\lambda \;\cdot \;\frac{\left(1-{\text{e}}^{-\frac{\text{ST}}{{\text{T}}_{1\text{t}}}}\right)\;\cdot {\text{ e}}^{\frac{\text{PLD}}{{\text{T}}_{1\text{b}}}}}{2\;\cdot \;\varepsilon \;\cdot \left(1-{\text{e}}^{-\frac{\text{LT}}{{\text{T}}_{1\text{b}}}}\right)}\;\cdot \;\left(\frac{\text{PW}}{{\text{SF}}_{\text{PW}}\;\cdot \text{PD}}\right)$$

where T1 of blood (T_1b_) was assumed to be 1.6 s at 3.0T, T1 of tissue (T_1t_) = 1.2 s, partition coefficient (λ) = 0.9, labeling efficiency (ε) = 0.6, saturation time of PD (ST) = 2s, labeling duration (LT) = 1500 ms, and post-labeling delay (PLD) = 2525 ms. PW is the perfusion weighted or the raw difference image; PD is the partial saturation of the reference image and SF_PW_ is an empirical scaling factor (= 32) used to increase the dynamic range of the PW [15].

**Fig 1:**
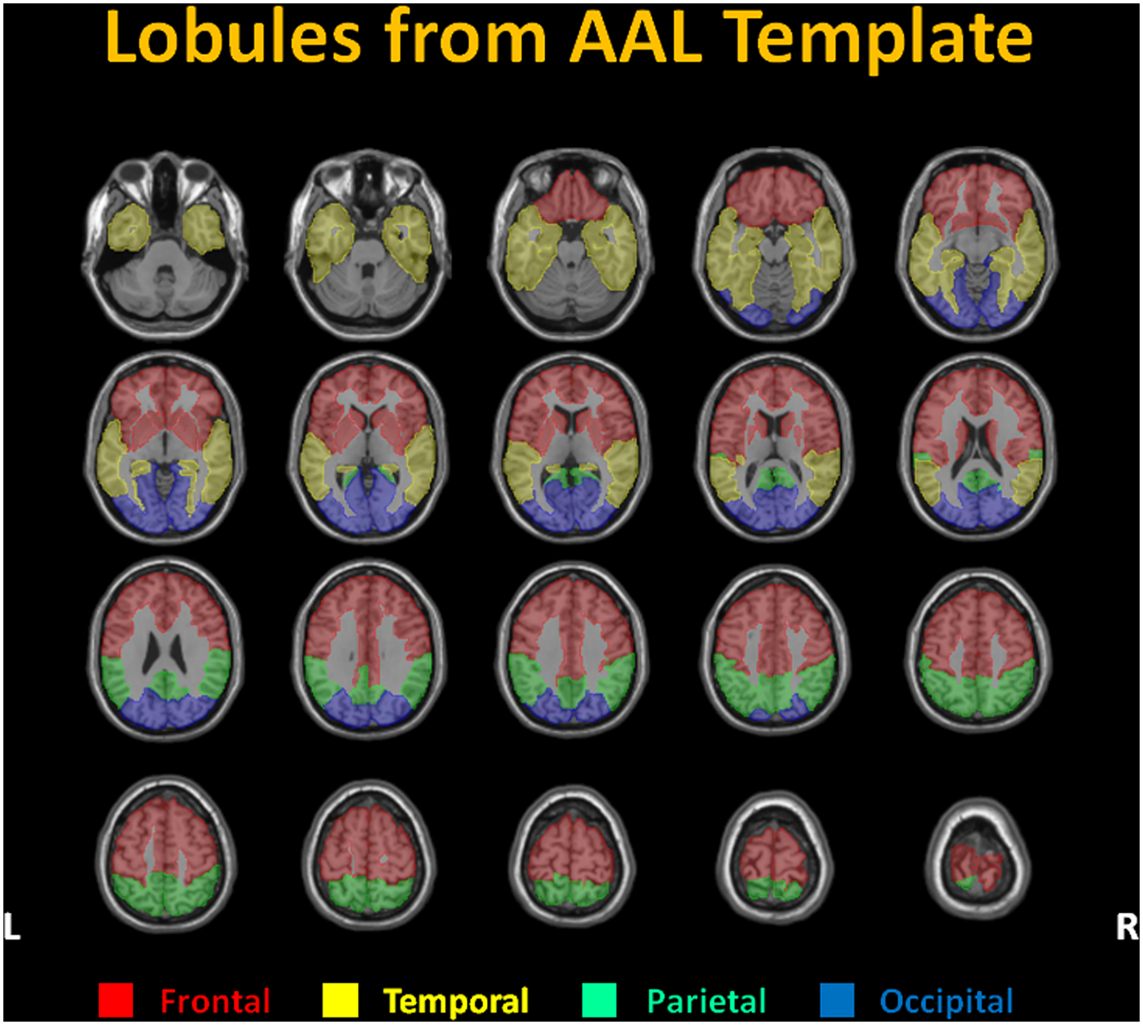
Automated anatomical labeling (AAL) template for MR data analysis. Cerebral cortex in respective lobes including frontal, parietal, temporal, and occipital lobes was encoded with different colors on the AAL template excluding the white matter and ventricles. The average cortical CBF in each lobe derived from the respective lobular ROI.

Images analysis was implemented using SPM8 (Statistical Parametric Mapping 8; Wellcome Department of Imaging Neuroscience, London, UK; http://www.fil.ion.ucl.ac.uk/spm/) [16]. Firstly, the high-resolution T1-weighted images were coregistered to ASL images of the same patient using a 3D rigid-body registration. Based on the coregistered T1-weighted images, the predefined regions of interest (ROI) in Montreal Neurological Institute space were then spatially normalized into the native space individually for each subject. A predefined set of ROIs in MNI space included the bilateral frontal, parietal, temporal, and occipital lobes, as well as the bilateral anterior cerebral artery (ACA), middle cerebral artery (MCA), and posterior cerebral artery (PCA) territories. The lobular ROIs were defined based on the automated anatomical labeling (AAL) template [17] shown in Fig 1 and examined by an experienced neurologist. The territory ROIs were derived from literature [18]. For each subject, the average CBF values (expressed in milliliters per 100 grams of tissue per minute, mL/100g/min) were extracted by averaging the CBF values within each ROI. Finally, comparison of regional CBF values between normal controls and MTBI patients for each ROI was carried using two-sample t-test. The significant between-group comparisons were determined based on a statistical threshold of two-tailed, uncorrected *p* < 0.05. Regression analysis was performed between average CBF and neuropsychological tests in MTBI patients by Spearman's rank correlation analysis and was statistically significant if *p* < 0.05.

### Region of interest analysis

The average beta values of CBF were calculated within the bilateral ACA, bilateral MCA and bilateral PCA ROIs. The significant between-group (control and MTBI) was determined based on a statistical threshold of two-tailed, uncorrected *p* < 0.05. The average CBF with ROIs were used in regression analyses and correlated with neuropsychological test results on the regions showing remarkable differences between the normal controls and MTBI patients.

## Result

Patient demographics and clinical data are summarized in the Table 1. There were 23 MTBI patients (7 males and 16 females) and 22 normal controls (5 males and 17 females). The mean age of MTBI patients was 51.6 ± 6.73 years, whereas that of the normal controls was 52.1 ± 9.7 years. No statistical significance of the age and sex between the MTBI patients and normal controls. Twenty-three patients at initial visit had Glasgow Coma Scale score of 15 at the emergency department. The causes of trauma in the 23 patients included motor vehicle collisions (n = 11), falls (n = 8), hit by objects (n = 2), spots injury (n = 1) and assault (n = 1).

**Table 1:** Demographic data in normal controls and MTBI patients.

	Normal controls (n = 22)	MTBI patients (n = 23)	*p*-value
Sex, male / female	5 / 17	7 / 16	-
Age, year	52.1 ± 9.7	51.6 ± 8.41	0.83
Days since injury at baseline	-	12.57 ± 4.13	-
GCS score at baseline	15	15	-

Abbreviations: GCS = Glasgow Coma Scale. MTBI = mild traumatic brain injury.

All the patients received the initial MRI and neuropsychological tests at the subacute stage of MTBI (12.57 ± 4.13 days).

Quantitative results of average CBF values in the lobular regions and territories presented in the Table 2 and revealed CBF reduction in the MTBI patients as compared with the normal persons. Conventional MR imaging scans showed no structural abnormalities observed in any of the MTBI patients and controls. Average CBF was quantified as hypoperfusion in the left frontal lobe of 49.62 ± 8.41 mL/100g/min (p = 0.02) as compared with that of the normal controls (54.87 ± 6.54 mL/100g/min). The average CBF of the MTBI group in the right frontal lobe is 49.42 ± 8.64 mL/100g/min (p = 0.04) as comparing with the normal persons (54.25 ± 6.89 mL/100g/min). The average CBF of the MTBI group in the left occipital lobe is 48.32 ± 9.3 mL/100g/min (p = 0.03) as comparing with the normal persons (54.27 ± 7.97 mL/100g/min).

**Table 2:** Quantitative results of average CBF values in normal controls and MTBI patients.

	Normal controls (n = 22)	MTBI patients (n = 23)	*p*-value
Average CBF values in the lobular regions			
Frontal lobe			
Left	54.87 ± 6.54	49.62 ± 8.41	0.02*
Right	54.25 ± 6.89	49.42 ± 8.64	0.04*
Parietal lobe			
Left	55.92 ± 7.98	50.73 ± 9.87	0.06
Right	54.71 ± 8.97	50.68 ± 10.06	0.16
Temporal lobe			
Left	52.77 ± 6.77	48.58 ± 8.32	0.07
Right	52.85 ± 8.41	47.91 ± 8.72	0.06
Occipital lobe			
Left	54.27 ± 7.97	48.32 ± 9.3	0.03*
Right	55.06 ± 7.58	50.93 ± 10.43	0.14

Significant (*p* < 0.05) cortical CBF reduction among the subacute MTBI patients located in the bilateral frontal and left occipital lobes as compared with normal controls’ CBF.

* *p* < 0.05, statistically significant

Abbreviations: CBF = Cerebral blood flow.

For understanding the neuropsychological impairment in MTBI group, the mean scores and standard deviation on the six neuropsychological tests including BAI, DHI, SSQ, DS, CPT-II and WCST-perseverative error (WCST-P) with the score of the each subcategory in the 23 MTBI patients are summarized in Table 3. In the MTBI patients, dizziness, simulator sickness, anxiety and perseverative errors of executive function were pronounced. In the BAI, DHI and SSQ, the higher score indicated the more severe deficits. In the WCST-P, the lower score means the poor executive function with respect to the perseverative errors.

**Table 3:** Neuropsychological tests of the MTBI patients.

**DHI**	**Mean** ± **SD**
Total	35.3 ± 27.92
Functional	14.26 ± 12.64
Emotional	9.65 ± 9.0
Physical	11.39 ± 7.85
**SSQ**	**Mean** ± **SD**
Total	381.35 ± 391.6
Nausea	19.5 ± 24.49
Oculomotor	35.26 ± 27.86
Disorientation	47.21 ± 57.51
**DS**	**Mean** ± **SD**
Forward, raw	14.43 ± 1.62
Forward, scaled	12.87 ± 1.46
Backward, raw	7.57 ± 3.26
Backward, scaled	11.3 ± 3.18
Total, raw	22.0 ± 4.32
Total, scaled	12.52 ± 2.69
**BAI**	**Mean** ± **SD**
Total	10.17 ± 9.67
**CPT II**	**Mean** ± **SD**
Omissions	46.05 ± 14.28
Commissions	49.31 ± 4.18
Hit RT	61.86 ± 3.65
**WCST-P**	**Mean** ± **SD**
Perseverative Errors	49.1 ± 2.99

Mean and standard deviation (SD) in six neuropsychological tests on dizziness, simulator sickness, attention, anxiety, and executive function were obtained from 23 MTBI patients within 1 month.

Abbreviations: DHI = Dizziness Handicap Index; SSQ = Simulator Sickness Questionnaire; DS = Digit Span; BAI = Beck Anxiety Inventory; CPT II = Conner's Continuous Performance Test II; WCST-P = Wisconsin Card Sorting Test- perseverative errors.

The clinical correlations between the CBF values within affected hypoperfused lobes of the MTBI patients and the neuropsychological impairments are shown in Table 4. The CBF in the left frontal lobe correlated with DHI (*ρ* = 0.47, *p* = 0.02) and SSQ (*ρ* = 0.51, *p* = 0.01). The CBF in right frontal lobe and left occipital lobe also correlated with SSQ (*ρ* = 0.45, *p* = 0.03 and *ρ* = 0.44, *p* = 0.04 respectively).

**Table 4:** Regression analysis of neuropsychological tests and CBF values in the MTBI patients.

	Frontal lobe		Parietal lobe		Temporal lobe		Occipital lobe	
	Left	Right	Left	Right	Left	Right	Left	Right
**DHI**								
Total	**0.47** *	0.39	0.34	0.24	**0.52** *	0.33	0.37	0.25
Functional	0.54 *	0.47*	0.42	0.34	**0.59** **	0.40	**0.43** *	0.34
Emotional	0.41	0.34	0.28	0.21	**0.45** *	0.29	0.32	0.21
Physical	0.31	0.26	0.22	0.16	0.39	0.23	0.27	0.17
**SSQ**								
Total	**0.51** *	**0.45** *	**0.44** *	0.36	**0.55** *	0.42	**0.44** *	0.35
Nausea	**0.50** *	0.40	0.41	0.27	**0.50** *	0.30	0.39	0.31
Oculomotor	**0.54** *	**0.48** *	**0.44** *	0.35	**0.55** *	0.40	**0.43** *	0.35
Disorientation	**0.44** *	0.40	0.36	0.32	**0.51** *	0.41	0.39	0.30
**BAI**								
Total	0.40	0.35	0.28	0.28	**0.46** *	0.37	0.35	0.31
**WCST**								
Perseverative Errors	0.31	0.33	0.33	0.39	0.42	**0.46** *	**0.46** *	0.42

*: *p* < 0.05;

**: *p* < 0.01;

Abbreviations: DHI = Dizziness Handicap Index; SSQ = Simulator Sickness Questionnaire; BAI = Beck Anxiety Inventory; WCST-P = Wisconsin Card Sorting Test- perseverative errors.

**Fig 2:**
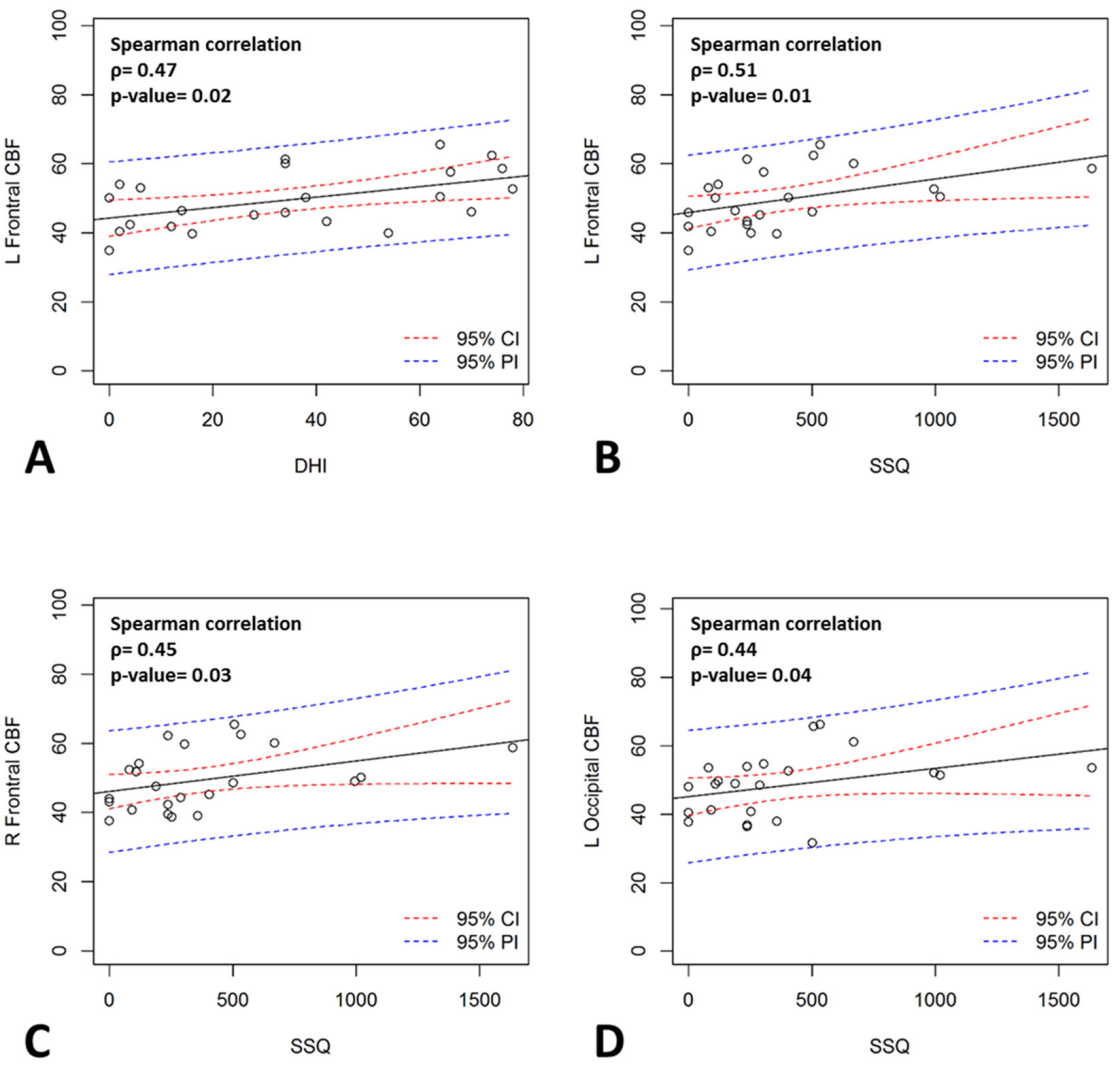
Correlation of CBF in bilateral frontal and left occipital lobes with DHI and SSQ at the subacute stage of MTBI. (A)Positive correlation between the averaged CBF at left frontal lobe with DHI. (B)Positive correlation between the averaged CBF at left frontal lobe and SSQ. (C)Positive correlation between the averaged CBF at right frontal lobe and SSQ. (D)Positive correlation between the averaged CBF at left occipital lobe and SSQ.

In summary, MTBI group had lower CBF in bilateral frontal and left occipital lobes and CBF values in left frontal cortical gray matter showed significant correlation with dizziness and simulator sickness (Fig 2A and 2B). Also, CBF in right frontal cortex and left occipital cortex both correlated with simulator sickness (Fig 2C and 2D). CBF was not correlated with BAI, WCST-P, DS, and CPT-II.

## Discussion

Regional reduction in CBF has been previously documented in patients in the early stages following TBI using either stable xenon-enhanced CT [19–24] or SPECT [25–27]. The current study indicated the regional reduction of CBF can also be detected at the subacute stage of MTBI by using ASL technique, which may suggest presence of brain injury in affected regions.

In addition to the average CBF reduction in the bilateral frontal lobes and left occipital lobes among the subacute MTBI patients, there were positive correlation between CBF value and post concussive symptoms including dizziness and simulator sickness. Our result showed that higher CBF corresponded to worse symptoms in those areas of hypoperfusion. However, we could not provide a good explanation to this phenomenon based on this preliminary study.

The neuropsychological deficits possibly correlated to the unstable cortical CBF status due to the contact injury mechanism of the MTBI. Most functional image studies suggest that functional cerebral abnormalities, mostly CBF reduction, exist in the chronic phase on CT perfusion, SPECT, PET, perfusion MRI [28]. Contact mechanism may resulted in the post-MTBI cortical hypoperfusion due to direct damage and disrupted integrity of the neuron and gliovascular unit with initiation of the coagulation cascade resulting in CBF reduction [29, 30]. Not only the shearing force to the deep white matter at thalamus related with the CBF reduction [31] but also the direct contact injury had a role in the cortical CBF reduction.

In the current study, our CBF measurements were performed on approximate 20 days after the injury, implying that hemodynamic change may be detected at suspected injured cerebral cortex in subacute stage. In addition, the presence of correlation between CBF and post concussive symptoms, including dizziness and simulator sickness, suggested that change in cerebral hemodynamics may play a role in pathophysiology underlies the symptoms.

In the clinical management of subacute or chronic MTBI with persistent symptoms or deficits, MRI had a role in the American College of Radiology guidelines [32]. With the advent of modern MR brain image technique and availability of commercial software, ASL not only provides additional quantitative evaluation of cerebral hemodynamics without radiation or contrast medium administration, but also facilitates longitudinal post-MTBI follow up with reproducible measurement.

There are some limitations in our study. First, small sample size did not allow extensive analysis. Second, no neuropsychological tests were performed in the normal controls. Our study results are preliminary. Future study with larger sample size and follow up with combination of other modalities such as diffuse tensor image, functional MRI study, and susceptibility-weighted may help to explore underlying pathophysiology of MTBI.

## Conclusion

First, this study determined that despite no significant abnormality detected on conventional CT and MRI studies, hypoperfusion was observed in MTBI group using 3D-PCASL technique in subacute stage, which suggested that this approach may increase sensitivity to MTBI. Second, the correlation between CBF and the severity of post concussive symptoms suggested that changes in cerebral hemodynamics may play a role in pathophysiology underlies the symptoms.
